# *Sprouty2* mediated tuning of signalling is essential for somite myogenesis

**DOI:** 10.1186/1755-8794-8-S1-S8

**Published:** 2015-01-15

**Authors:** Muhammad Abu-Elmagd, Katarzyna Goljanek Whysall, Grant Wheeler, Andrea Münsterberg

**Affiliations:** 1 Center of Excellence in Genomic Medicine Research (CEGMR), King Abdulaziz University, P.O. Box: 80216, Jeddah 21589, Kingdom of Saudi Arabia; 2 KACST Center of Innovation in Personalized Medicine (CIPM), King Abdulaziz University, P.O. Box: 80216, Jeddah 21589, Kingdom of Saudi Arabia; 3 Zoology Department, Faculty of Science, Minia University, El- Minia, P.O. Box 61519, Egypt; 4 Department of Musculoskeletal Biology, Institute of Ageing and Chronic Disease, Faculty of Health and Life Sciences, University of Liverpool, UCD Building, Daulby Street, Liverpool L69 3GA, United Kingdom; 5 Department of Cell and Developmental Biology, School of Biological Sciences, University of East Anglia, Norwich, NR4 7TJ, United Kingdom

**Keywords:** Somite myogenesis, *FDF/Spry2* signalling, proliferation, negative regulation

## Abstract

**Background:**

Negative regulators of signal transduction cascades play critical roles in controlling different aspects of normal embryonic development. *Sprouty2* (*Spry2*) negatively regulates receptor tyrosine kinases (RTK) and FGF signalling and is important in differentiation, cell migration and proliferation. In vertebrate embryos, *Spry2* is expressed in paraxial mesoderm and in forming somites. Expression is maintained in the myotome until late stages of somite differentiation. However, its role and mode of action during somite myogenesis is still unclear.

**Results:**

Here, we analysed chick *Spry2* expression and showed that it overlaps with that of myogenic regulatory factors *MyoD* and *Mgn*. Targeted mis-expression of *Spry2* led to inhibition of myogenesis, whilst its C-terminal domain led to an increased number of myogenic cells by stimulating cell proliferation.

**Conclusions:**

*Spry2* is expressed in somite myotomes and its expression overlaps with myogenic regulatory factors. Overexpression and dominant-negative interference showed that *Spry2* plays a crucial role in regulating chick myogenesis by fine tuning of FGF signaling through a negative feedback loop. We also propose that mir-23, mir-27 and mir-128 could be part of the negative feedback loop mechanism. Our analysis is the first to shed some light on in vivo *Spry2* function during chick somite myogenesis.

## Background

In early vertebrate embryos, including the chick, somites form as paired structures from unsegmented paraxial mesoderm on either side of the neural tube. Cells in the ventral somite undergo an epithelial to mesenchymal transition (EMT) to form the sclerotome [1]. The dorsal part remains epithelial forming the dermomyotome, which generates the epaxial and hypaxial domains of the myotome. The dermomyotome gives rise to the dermis and skeletal musculature whilst the sclerotome mainly gives rise to the ribs [2]. A network of signalling pathways and transcription factors coordinates the process of somite patterning and differentiation and how the myogenic programme in particular is activated differs in different parts of the body [3]. Sonic hedgehog (*Shh*) from the notochord and floor plate together with neural tube derived Wnt signals specify the epaxial dermomyotome which is important for generating the axial back muscles [4-6]. Wnt signals from the dorsal ectoderm and bone morphogenetic protein (BMP) signals from the lateral plate mesoderm promote formation of the hypaxial myotome, which generates the limb, diaphragm and body wall muscles [7]. Fibroblast growth factors (FGF) and extracellular signal-regulated kinases/mitogen-activated protein kinases (ERK/MAPK) have been found to be crucial during somite formation and in regulating limb myogenesis [8,9]. Downstream of FGF are Sprouty proteins, cytoplasmic membrane-associated proteins, which function by inhibiting receptor tyrosine kinase (RTK) signalling (reviewed in [10]). Sprouty was first identified in Drosophila by genetic screens a*s* an antagonist of the FGF receptor Breathless during tracheal branching [11].

To date, four mammalian homologs of *Drosophila* Sprouty have been identified (*Spry1-4*). They encode 32 to 34 kDa proteins that share a highly conserved carboxy-terminal cysteine-rich Sprouty domain and were shown to function as negative regulators of RTK signalling in vivo [12].

Sprouty transcripts showed highly restricted expression patterns during the morphogenesis of various embryonic tissues including limbs [13], lung [14], inner ear [15], kidney [16], testis [17], and tooth [18].

Functional studies implicate Sprouty proteins in the control of proliferation, cell migration, tracheal branching and angiogenesis [11,13,19-23]. It has been reported that human *SPRY2* triggered migration and proliferation of vascular smooth muscle cell and its expression increased in rat carotid artery injury model. This was associated with an inhibition of FGF signals and a decrease of proliferation [24]. In a cancer mouse model, loss of *Spry2* function led to an increase in B-cell proliferation due to hyperactivation of ERK/MAPK signalling [23]. The expression of FGF target genes was enhanced in palate of *Spry2* knockout mice, and loss of *Spry2* was associated with an increase in palate mesenchymal cell proliferation [25]. Recently, it has been shown that inhibiting *Spry2* expression in renal cell carcinoma promotes proliferation and invasion highlighting thus a potential role for *Spry2* during tumorigenesis [26].

We previously showed that chick *Spry2* is expressed in developing somites. In particular, its transcripts were detected along the anterior and posterior somite edges and in the centre of mature myotomes in a thin stripe suggesting a possible function in secondary myogenesis and myotome growth [9]. During limb bud development, it has been reported that *Spry2* and *Spry1* are expressed in muscles and tendons in both chick and mouse. In *Pax3* knockout mice Sprouty expression was lost indicating that they are expressed in muscle progenitors [27]. In addition, it has been shown, by using artificial regeneration and rescue experiments in mouse, that FGF6 and *Spry2* are particularly involved in myogenesis [28]. Overexpression of *SPRY2* in C2C12 cells in presence of FGF2 led to induction of myogenesis whilst inhibition of *SPRY2* function led to myoblasts growth and failure of myotube formation. These results were the first evidence of *Spry2* playing a role during myogenic differentiation in presence of FGF2 in vitro [29].

Here we investigated the role of *Spry2* during somite myogenesis. We examined the regulation of *Spry2* in response to FGF and we analysed its expression compared to the myogenic markers *MyoD* and *Mgn* using double *in situ* hybridisation. Functional interference approaches utilized targeted mis-expression by electroporation showed that *Spry2* inhibits somite myogenesis. In contrast, inhibition of *Spry2* function using its C-terminal interference promoted somite myogenesis by increasing proliferation of myogenic cells in the dermomyotome and myotome. Our results indicate that *Spry2* regulates chick somite myogenesis through a negative feedback loop to FGF2 and other factors including microRNAs could be playing a role in this mechanism.

## Results and discussion

### *Sprouty2* expression during somite myogenesis

**Figure 1:**
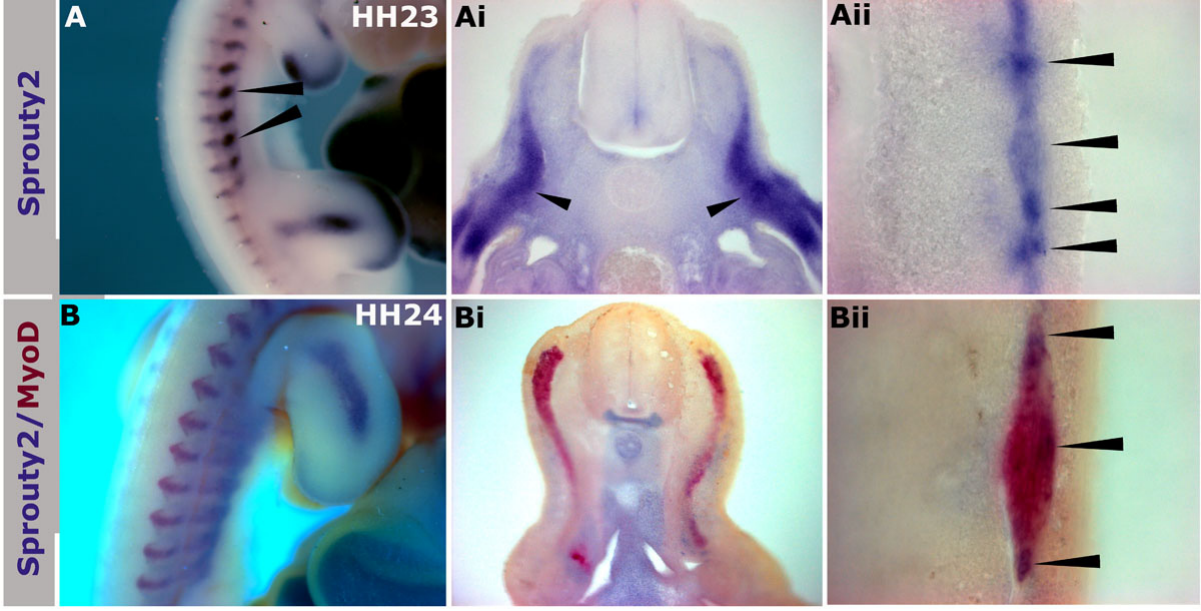
**Expression of *Spry2* in somites and limb buds is closely associated with *MyoD*.** (A) Whole mount *in situ* hybridisation of chick embryo at HH23 showing *Spry2* expression in limb buds and somites, with strong signal visible in the hypaxial domains (arrowheads). (Ai) Transverse section of embryo in (A) at the level of trunk somite showing *Spry2* expression throughout the myotome with high levels in the hypaxial domain (arrowheads). (Aii) Frontal section shows *Spry2* expression in the myotomes, anterior and posterior myotome edges are indicated by arrowheads. (B-Bii) Double *in situ* hybridisation detects *Spry2* (purple, NBT/BCIP) and *MyoD* (red, Fast red) at HH24, (B) whole mount chick embryo shows overlap in somites and limb bud, (Bi) transverse and (Bii) frontal sections show overlapping expression of *Spry2* and *MyoD*. Magnifications: 20x in (A), 200x in (Ai), 400x in (Aii & Bii), 22x in (B), 100x in (Bi).

*Spry2* expression during somite development was analysed at different Hamburger and Hamilton (HH) stages. At stage HH11 expression was restricted to the pre-segmented paraxial mesoderm (PSM) and in somites expression was very weak (data not shown). Between HH20-HH27 expression was detected throughout the myotome and signal was especially strong in the hypaxial domain (Figure 1A-Ai). Frontal sections through the somites confirmed expression in the myotome with increased signal strength at the myotome boundaries at the junctions with the syndetome, containing tendon progenitors (Figure 1Ai, Aii, arrowheads). In addition to its expression in somites, *Spry2* was expressed in both forelimb and hindlimb buds, in the mesenchyme just beneath the apical ectodermal ridge and in developing limb muscle (Figure 1A and Additional file Figure S1A, Ai). Double *in situ* hybridisation with the myogenic markers, *MyoD* and *Mgn*, showed clearly a high degree of overlap (Figure 1B-Bii and Additional file Figure S2A-Aiii).

We have previously detected *Spry2* transcripts in developing chick somites [9]. Here, we further analysed these findings by co-localising *Spry2* expression with that of the early myogenesis markers *MyoD* and *Mgn*. Our results are consistent with other previously reported localisation of the mouse *Spry2* transcripts in the myotomes and dermomyotomes [27]. In addition, a number of studies have also highlighted the involvement of Spry1 and Spry2 proteins during the myogenic differentiation [28,29].

### *Sprouty2* expression is regulated by FGF2

**Figure 2:**
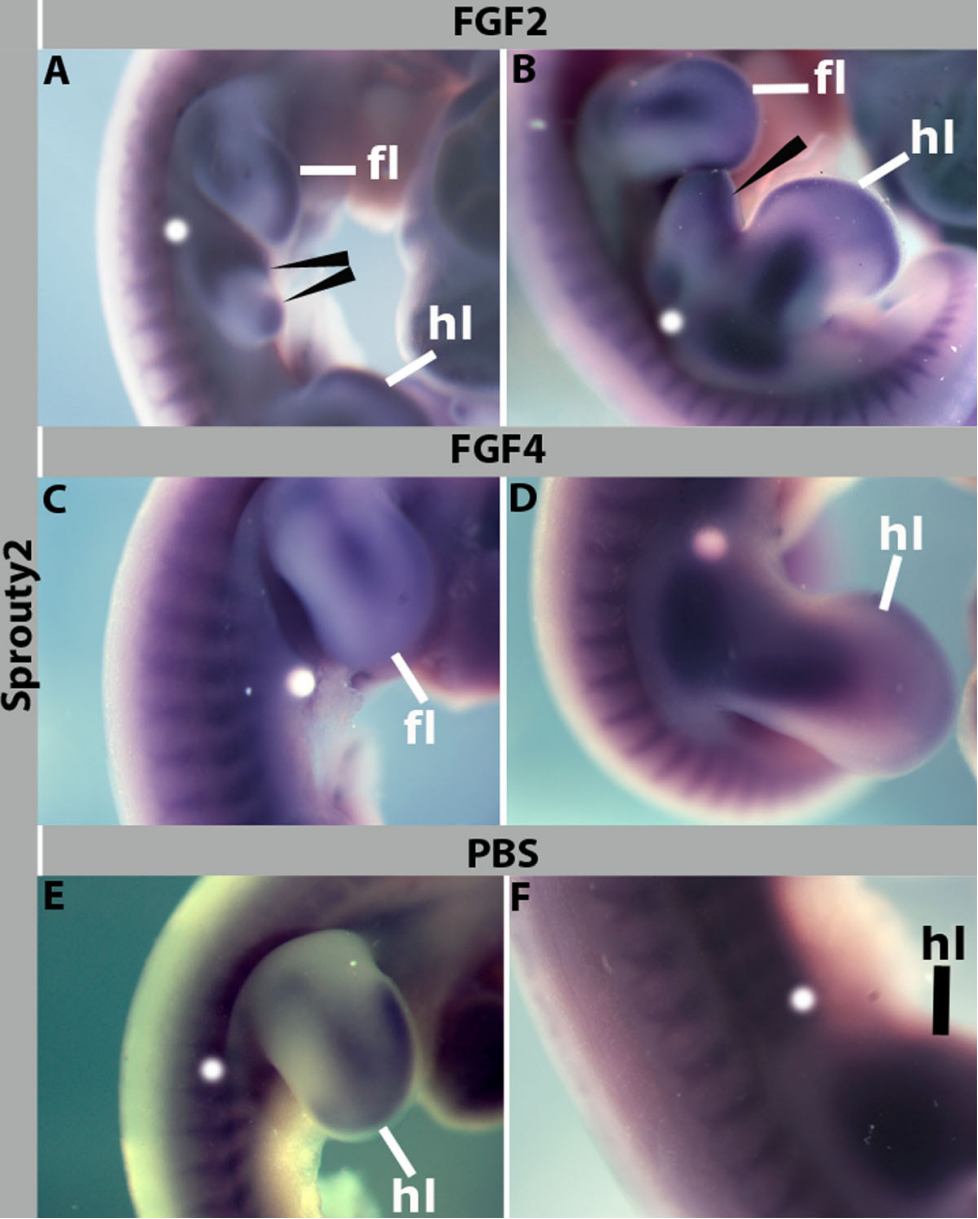
**FGF2 activates ectopic *Spry2* expression.** (A, B) FGF2 beads implanted adjacent to flank level somites of HH16 embryos induced outgrowth of an ectopic limb bud with *Spry2* expression near the tip (arrowheads), *Spry2* expression is expanded at the base of the forelimb (in A, arrowhead) or hindlimb bud (in B) towards the bead. (C, D) FGF4 beads implanted adjacent to flank level somites of HH16 embryos do not induce ectopic *Spry2* expression. (E, F) PBS control beads implanted similarly to that of FGF2 and FGF4 at the level of the forelimb (E) or hindlimb (F) flanks and show normal *Spry2* expression. fl: Forelimb, hl: hindlimb. Magnifications: 18x in (A, B), 22x in (C, D, E), 24x in (F).

In many tissues the expression of *Spry2* is activated by FGFs and it acts in a negative feedback loop as an inhibitor of FGF signalling (reviewed in [12]). To determine whether FGF can regulate *Spry2* in paraxial and lateral plate mesoderm, we implanted beads soaked with FGF2 (400μg/ml) or FGF4 (50μg/ml) adjacent to forelimb or hindlimb and flank level somites of HH16 embryos. PBS beads were used as a control. Embryos were allowed to develop for 24 hours and *Spry2* expression was analysed by *in situ* hybridization. In embryos implanted with FGF2 beads an ectopic limb bud was induced (Figure 2A, B, arrowheads, 100%, n=15). This was associated with *Spry2* expression in the distal tip of the ectopic limb bud, similar to the normal limb. In 50% of embryos we observed an increase of *Spry2* expression in the mesoderm close to the bead (Figure 2A, arrowhead). Interestingly, when FGF4 soaked beads (50μg/ml) were implanted similarly to FGF2, no ectopic limb buds or increased Spry2 was detected (n=9)(Figure 2C, D). This may indicate that the effect of FGF2 beads is specific, however we cannot exclude the possibility that higher concentrations of FGF4 may have the same effect. Control PBS beads did not affect *Spry2* expression (n=8) (Figure 2E, F).

It has been reported previously that *Spry2*, as well as other related proteins such as Spred and Sef, are positively regulated by FGF signalling [30]. The first evidence suggesting the involvement of FGF2 and *Spry2* in myogenic differentiation through a negative feedback loop was obtained in a well characterized cell based system using C2C12 myoblasts [29]. However, in chick limb mesenchyme, it has been reported that FGF4 rather than FGF2 positively regulated *Spry2* expression [27]. In the context of neural development, *Spry2* downregulation resulted in upregulation of FGF2 and promoted axonal elongation [31].

### Targeted misexpression of *Sprouty2* affects somite myogenesis

**Figure 3:**
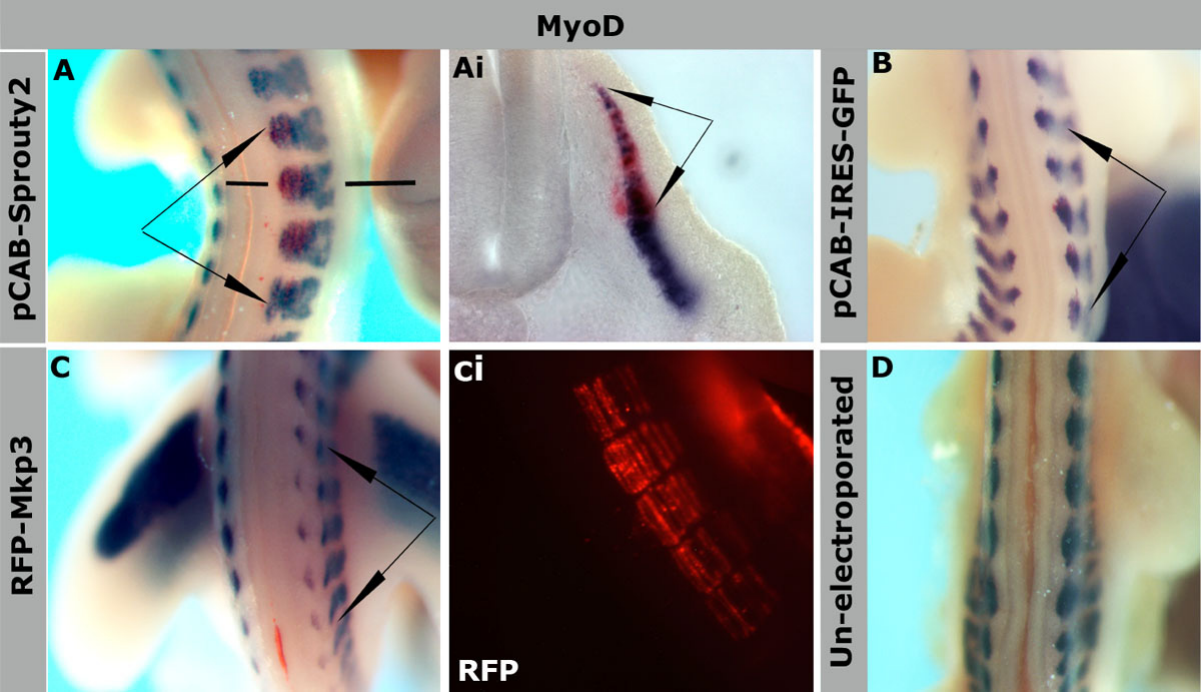
***Spry2* gain-of-function inhibits somite myogenesis.** Electroporation of pCAB-*Spry2* full length or RFP-*MKP3* expression constructs into epithelial somites at HH16, as indicated, followed by 24-hour incubation to HH21/22. (A-C) Whole mount *in situ* hybridisation for *MyoD* (purple) and GFP or RFP (red), (Ai) is a section of embryo in (A). (A, Ai) *Spry2* expression led to loss of *MyoD* in transfected cells, arrows in (A) indicate the targeted electroporated somites and lines indicate the level of sectioning in (Ai). (B) Electroporation of an empty pCAB-IRES-GFP expression vector (used as a control) into somites which showed normal *MyoD* expression (arrows). (C) Loss of *MyoD* was observed in cells electroporated with RFP-*Mkp3* (arrows); (Ci) RFP detected by fluorescent filter Alexa-Fluor-465 in the same electroporated somites in (C). (D) Control of unelectroporated embryo showing normal *MyoD* expression. Magnifications: 20x in (A, B, C & D), 24x in (Ci), 200x in (Ai).

**Figure 4:**
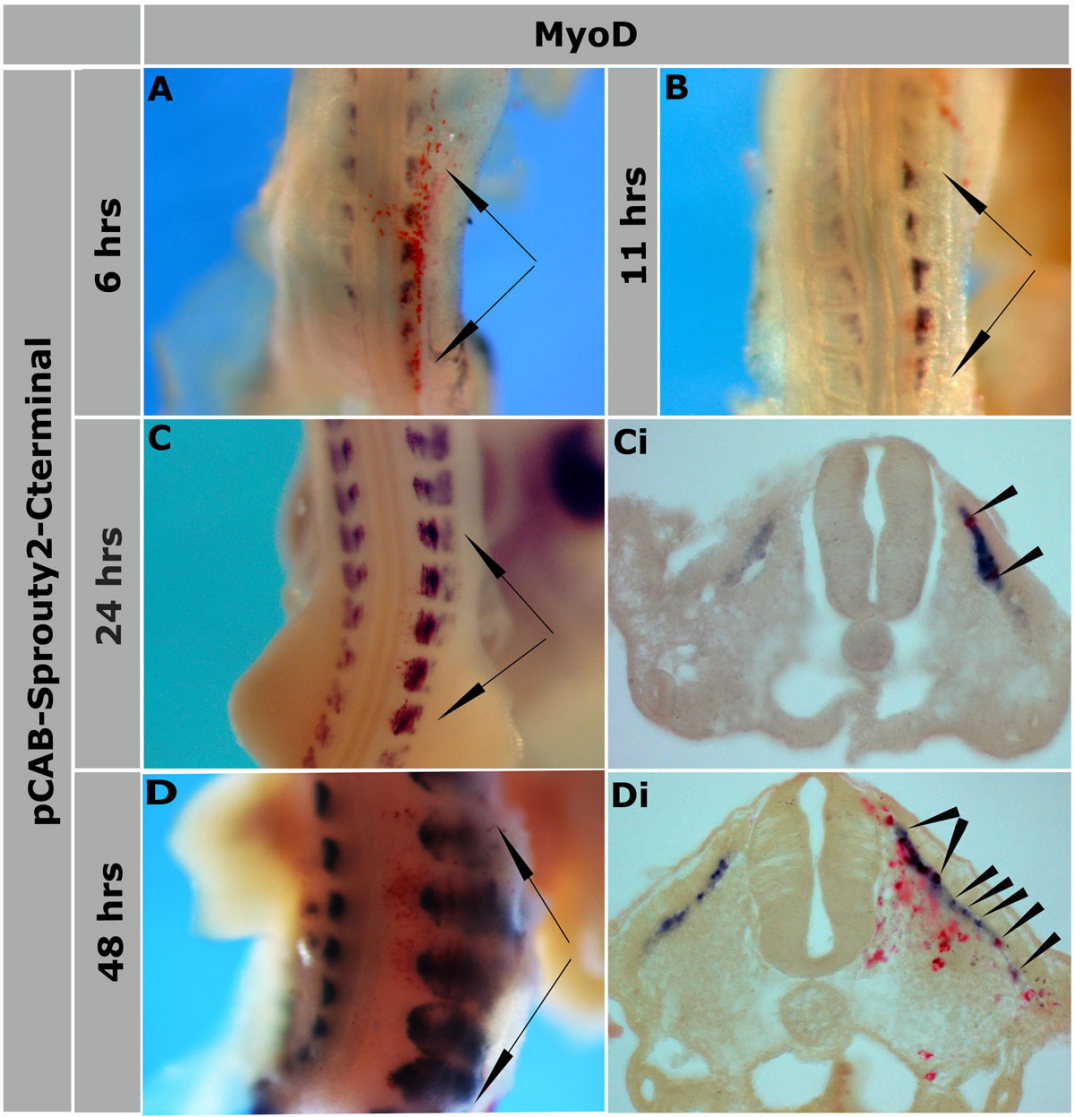
***Spry2*-Cterminus promotes somite myogenesis.** pCAB-*Spry2*-Cterm was overexpressed in epithelial somites by electroporation followed by harvesting embryos at different time points of incubation: (A) 6-hours, (B) 11-hours, (C, Ci) 24-hours and (D, Di) 48-hours. An increase of *MyoD* expression was detected (A-Di) which was associated with an increase in the somites size after incubation of 48-hours (D, Di). Arrows in (A, B, C & D) indicate the electroporated somites and arrowheads in (Ci, Di) indicate the electroporated myotomes and cells. Magnifications: 20x in (A, B, C & D), 200x in (Ci, Di).

Next we wanted to examine the role of *Spry2* in somite myogenesis using a gain-of-function and a functional interference approach. First, we used microinjection and electroporation of a plasmid encoding full length *Spry2* and GFP from the same vector backbone (pCAB-*Spry2*-IRES-GFP). Epithelial somites of HH16-17 embryos were targeted. Embryos were harvested 24- or 48-hours after electroporation after which transfected somites were identified by GFP fluorescence and effects on myogenesis were examined by analysing changes in *MyoD* expression using *in situ* hybridisation. This revealed a loss of *MyoD* expression in regions of somite transfected with pCAB-*Spry2*-IRES-GFP (n=19) (Figure 3A, Ai). There was no difference in the effect of *Spry2* misexpression on *MyoD* expression if electroporation was in dermomyotomes or myotomes. Control embryos electroporated with pCAB-IRES-GFP showed normal *MyoD* expression (Figure 3B). We compared this phenotype to that obtained with a different antagonist of FGF signalling, which we had previously characterized [9]. We electroporated expression constructs encoding *Mkp3*, a dual-specific phosphatase, which inactivates ERK. Consistent with previous observations the electroporation of *Mkp3*-RFP led to localized loss of *MyoD* expression in transfected somites (Figure 3C-Ci) (n=13, see also [9]). Un-electroporated control embryos showed normal *MyoD* expression (Figure 3D). Conversely, electroporation of a truncated form of *Spry2* which only contains the carboxy-terminus, led to promotion of myogenesis indicated by an increase in *MyoD* expression (n=27) (Figure 4). This was detectable after 6 hours of electroporation (Figure 4A). Increased expression of *MyoD* was also observed after 11, 24 and 48 hours (Figure 4B-Di). We also noticed an increase in somite size after longer incubation for 48 hours, when comparing electroporated somites with those on the opposite side (Figure 4D, Di).

It has been previously shown that a *Spry2* truncation, which consists of only the C-terminal part, leads to loss of *Spry2* function [32]. It is still incompletely understood how FGF signalling is regulated by its inhibitors during early somite development. A number of FGF negative regulators, among which is *Spry2*, have been identified (reviewed in [33]). Here, the manipulations of *Spry2* activity using gain-of-function and dominant-negative interference suggest that it affects FGF signaling, potentially activated by FGF2, through a negative feedback loop mechanism. It has been shown in a considerable number of other contexts that *Spry2* regulates FGF signalling through negative feedback. For example, this was demonstrated in the developing limb bud and neural plate [30], sensory neurons of dorsal root ganglia [31], 293 kidney cells [32], fibroblasts [34], brain [35], angiogenesis [36], neuritis in PC12 cells [37] and in Xenopus Spemann’s organizer [38].

### Inhibition of *Sprouty2* function increased the number of mitotic cells in dermomyotomes and myotomes

**Figure 5:**
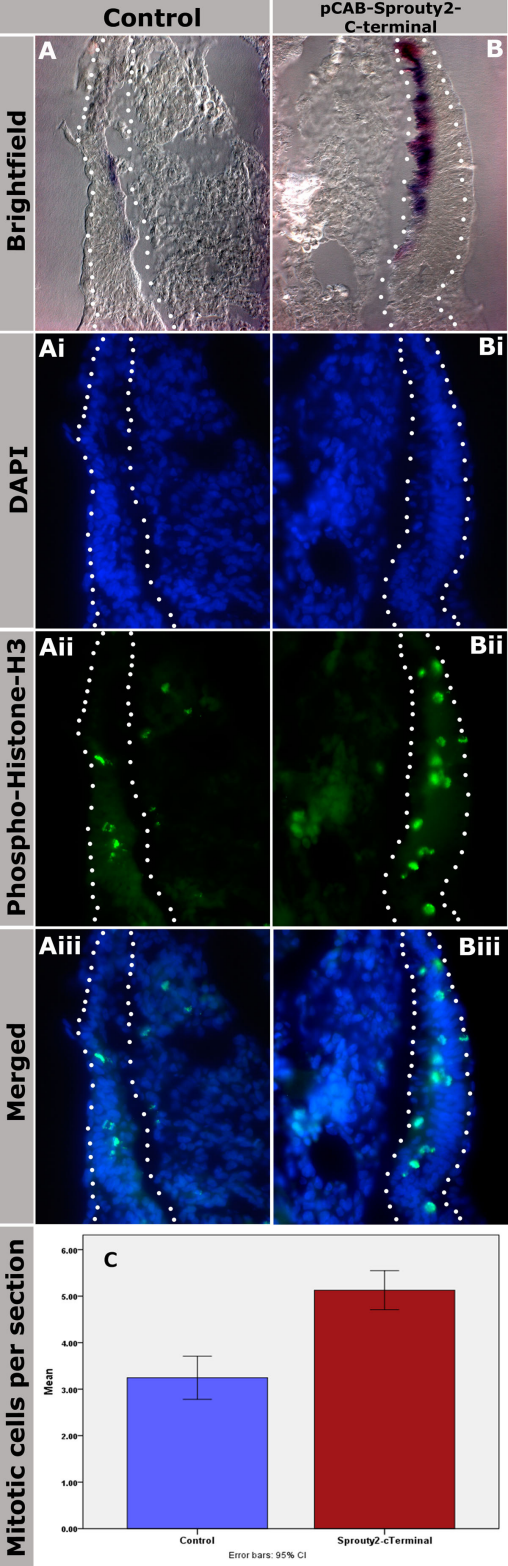
**Targeted misexpression of pCAB-*Spry2*-Cterminal results in increased number of mitotic cells in the dermomyotome and myotome.** (A-Aiii) Brightfield and fluorescent images of the electroporated control side. (B-Biii) Brightfield and fluorescent images of pCAB-*Spry2*-Cterminal injected and electroporated somites. (A, B) Increased *MyoD* expression was detected by *in situ* hybridization (purple) in the injected/electroporated side in (B) with GFP detected in red. (Ai, Bi) Sections were stained with DAPI and dermomyotome and myotome boundaries are indicated with dotted white lines. (Aii, Bii) Immunostaining with anti-Phospho-Histone-H3 detects mitotic cells and shows an increase of the number of the dividing cells in injected dermomyotome and myotome (Bii, within the dotted white lines) in comparison to the contralateral control somite in (Aii). (Aiii, Biii) merged images of DAPI in (Ai, Bi) and Phospho-Histone-H3 stained sections in (Aii, Bii). Maginfications: 200x in all sections. (C). Statistical analysis of the anti-Phospho-Histone-H3 proliferation assay for pCAB-*Spry2*-Cterminal. Columns represent results of the SPSS statistical analysis of the proliferating cells in myotomes and dermomyotomes of uninjected somites (blue column, control) and in pCAB-*Spry2*-Cterminal electroporated somites (red column). The analysis confirmed that the differences in the means of paired counting (n=94) of the proliferating myocyte cells were statistically significant (p < 0.002), the diagram shows a graphical representation of these data. Error bars represent the standard deviation [48].

As reported above, we observed that misexpression of *Spry2*-Cterm caused increased *MyoD* expression. To investigate the mechanism by which *Spry2* induces this effect and to determine whether this might be due to increased proliferation of myogenic cells, we checked *MyoD* expression and combined this with that of anti-phospho-histone-H3 by immunostaining. We quantified the number of mitotic cells 24 hours after electroporation and compared microinjected somites with their contralateral non-injected counterparts (n=94 paired readings)(Figure 5A-B). The boundary of the dermomyotome, myotome and sclerotome in each section was determined by staining with DAPI. We observed an increase in the number of mitotic cells in the injected dermomyotomes and myotomes (Figure 5B-Biii) compared with that of the control side (Figure 5A-Aiii). This suggests that cell proliferation is affected. The number of proliferating cells was analysed by the SSPS using Wilcoxon test to determine whether the increase was statistically significant or not. The analysis detected an increase of 58% (P < 0.002) (Figure 5C).

*Spry2* has been previously shown to regulate cellular proliferation and migration in different biological systems [20,39]. Interference with *Spry2* function by the C-terminal part, which is a conserved domain in Drosophila, chick and mammals, was found to inhibit proliferation and migration of HeLa cells indicating that *Spry2*-C-terminus is important for its biological function [20]. Similarly, we tested this function of *Spry2* in the myotomes and dermomyotomes using a *Spry2* mutant expression construct (pCAB-*Spry2*-Cterm) that contains only the C-terminus. We observed a significant increase in myocyte number after inhibiting *Spry2* function, indicating that it attenuates myogenic cell proliferation in the forming somite.

In smooth muscle cells, wild type *SPRY2* inhibits migration and proliferation [24] and downregulation of its function by mir-21 in cardiomyocytes, promotes proliferation and cellular outgrowths [40]. Similarly, in TGW neuroblastoma cells, a dominant negative form of *SPRY2* promoted cell proliferation [41] and depletion of *Spry2* expression in renal cell carcinoma led to enhanced proliferation [26]. In *Spry1-2* knockout mice, it was reported that *Spry2* promoted cellular proliferation and brain neurogenesis [42]. In addition to these studies, it has been shown that loss of *Spry2* function in splenic B-cells resulted in an increase of phosphorylated *ERK1/2* activity and this was associated with an increase in B cells proliferation [23]. Altogether, these studies suggested that *Spry2* functions to regulate FGF/ERK-MAPK siganlling during cellular proliferation through a negative feedback loop. This notion in general is consistent with our results since in our experiments interference with *Spry2* function led similarily to an increase in the myocyte cell number. Additional antagonists of the FGF/ERK-MAPK signalling include the dual specificity phosphatase *PYSTI/Mkp3*, which similar to *Spry2*, initiates a negative feedback loop found to be important during limb bud outgrowth and neural induction [30] and the differentiation of scleraxis positive progenitors in developing somites.

### FGF/*Sprouty2* signalling is a possible pathway to regulate microRNA expression during somite myogenesis

**Figure 6:**
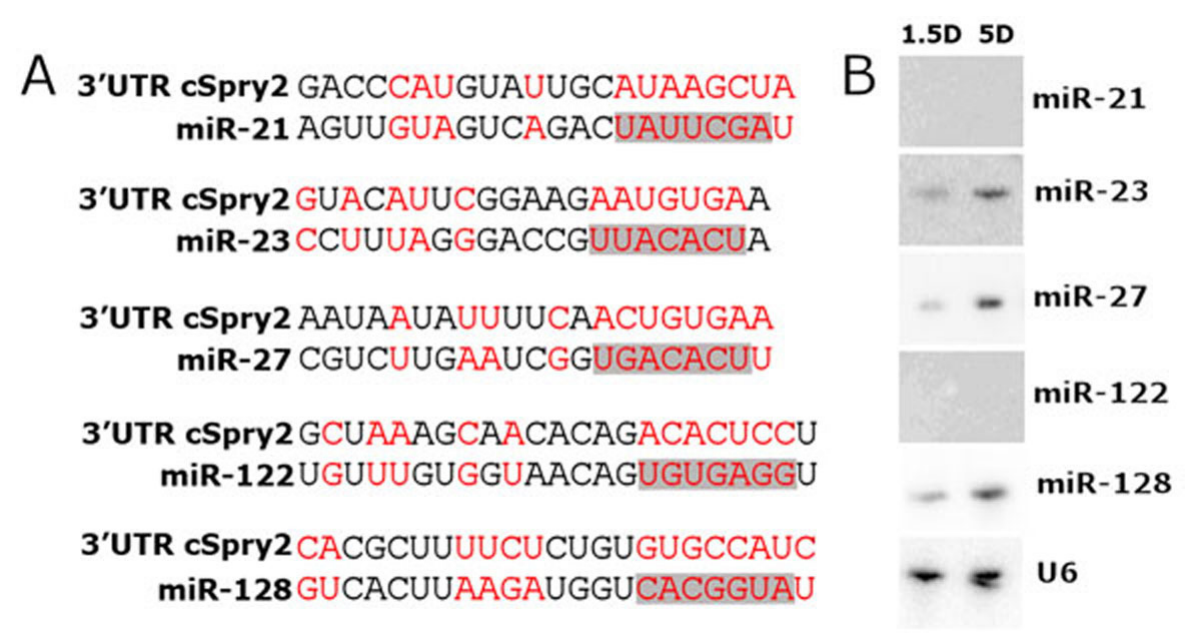
**Chick *Spry2* is a predicted target of a number of microRNAs.** (A). Sequence alignments show miR predicted targets sites within the 3’UTR of chick *Spry2* and chick microRNA sequences of *mir-21*, *mir-23*, *mir-27*, *mir-122*, and *miR-128*. Complementary nucleotides are highlighted in red and seed sequence of each microRNA is highlighted in grey. (B) Northern blot showing expression of the *microRNAs-21*, -*23*, *-27*, *-122* and *-128* in epithelial somites at HH10 (1.5 days) and HH27 (5-days) of chick embryo development, U6 nuclear snRNA was used as an internal control.

We previously showed that FGF signalling could regulate somite myogenesis by controlling microRNA expression [43,44]. FGF4 overexpression led to loss of *mir-206* indicating that it negatively regulates the initiation of *mir-206* gene expression [43]. In the current study, we used Targetscan (Version 5) to identify microRNAs predicted to target the 3’UTR of chick *Spry2*. The analysis showed that *mir-21*, *mir-23*, *mir-27*, *mir-122* and *mir-128* can potentially interact with *Spry2* through binding to its 3’UTR (Figure 6A). We next carried out Northern blotting of these microRNAs to check if they are expressed in epithelial somite RNA extracts at HH10 (1.5 Day) and HH27 (5 Day). In our hands, only *mir-23*, *mir-27* and *mir-128* showed strong expression in somites (Figure 6B). Together with the Targetscan analysis these results raise the possibility of the involvement of these three microRNAs in *FGF/Spry2* signalling. However, further analysis using gain- and loss-of-function of *mir-23*, *mir-27* and *mir-128* is required to proof or to exclude an interaction with *FGF/Spry2* signalling.

## Conclusions

We analysed the endogenous expression of *Spry2* during early somite myogenesis in chick and showed colocalisation of *Spry2* transcripts with two early myogenic markers *MyoD* and *Mgn*. In addition, we showed for the first time that overexpression of *Spry2* results in reduction of somite myogenesis indicated by loss of *MyoD* expression, and conversely interference with its function using its C-terminal domain resulted in promoting myogenesis by increasing the number of the myogenic cells. This suggests that *Spry2* could regulate myogenic cell proliferation activity in the forming somite in chick. We also demonstrated that FGF2 induces *Spry2* expression. It is also possible that other FGF ligands in addition to FGF2 could be tuned by *Spry2* via a negative feedback loop to regulate chick somite myogenesis including FGF4 and FGF8. Furthermore, additional players in *Spry2*/FGF negative feedback loop signalling may involve particular microRNAs. Our analysis of Targetscan followed by Northern blots of developing somites showed that *mir-23*, *mir-27* and *mir-128* could be part of this negative feedback loop mechanism.

## Materials and methods

### Embryo manipulations and electroporation

Fertile white Leghorn chicken eggs obtained from Henry Stuart (Lincolnshire) were incubated at 38°C and staged according to Hamburger and Hamilton (HH) [45]. Eggs were windowed and black ink was injected underneath the blastoderm to visualise the embryos. Electroporation was carried out as described previously [46]. Briefly, HH16 epithelial somites on one side of the embryo were injected with plasmid DNA at a concentration of 1-1.5 mg/ml. The pCAB-*Spry2* full length and pCAB-*Spry2*-Cterm (contains only the *Spry2* C-terminal part) plasmids were kind gifts from Professor Cornelis Weijer (University of Dundee). RFP-MKP3 construct was described in [9]. The contralateral, non-injected side was used as a control. Plasmids produced GFP or RFP, which allowed tracing of successful electroporation. Positive and negative platinum electrodes (0.3 mm diameter) were placed on either side of the somites, 5 pulses of 20 Volts for 35 milliseconds were applied using a TSS20 Ovodyne electroporator (Intracel). Eggs were sealed and incubated for 24, 48 hours or as indicated. After harvesting in DEPC/PBS, electroporated embryos were checked for GFP or RFP fluorescent signals using Leica dissecting microscope with fluorescent filters Alexa-Fluor-488 to detect GFP or Alexa-Fluor-465 to detect RFP. Only embryos showing positive signals in the targeted somites are processed for overnight fixation in 4% paraformaldehyde (4°C) and subsequent *in situ* hybridisation as described in [47].

### Bead implantation

The method described in [9] was followed. Briefly, Heparin beads (Sigma H-5263) were washed three times in PBS before soaking for 1 hour at room temperature in recombinant FGF (R&D Systems) at the following concentrations: FGF2 (400μg/ml) and FGF4 (50μg/ml). After washing twice in PBS, beads were implanted adjacent to forelimb or flank level somites of HH16 embryos. Control beads were soaked in PBS. Embryos were allowed to develop for 24 hours and were then analysed by whole mount *in situ* hybridisation.

### Probe synthesis, *in situ* hybridisation and immunostaining

Probes for whole mount *in situ* hybridisation were synthesized as described previously: [13] for *Spry2;*[30] for *Mkp3*; [9] for *MyoD* and *Mgn.* Single *in situ* hybridisation was carried out as previously described [5]. Double *in situ* hybridisation of *Spry2* (detected in blue or purple) and *MyoD* or *Mgn* (detected in red) was carried out as previously described [46].

For cryosectioning, embryos were fixed in 4% PFA/0.2% glutaraldehyde, washed with PBS, transferred to 30% sucrose/PBS and embedded in OCT. For immunostaining with anti-Phospho-Histone-H3 antibody (1:500, Developmental Studies Hybridoma Bank) sections (10 µm) were incubated with 0.1% Triton X-100 and treated with 1:10 H_2_O_2_/PBS for 10 minutes. After washing in PBS, sections were blocked in 10% goat serum and treated with primary antibody overnight at 4°C. Sections were blocked in 10% goat serum before applying secondary antibody (anti-rabbit fluorescent Alexa Fluor-488, 1:1000, Molecular Probes). Sections were treated with DAPI to stain nuclei and mounted in Mowiol. Pictures were taken with Axiovision software on an Axioscope (Zeiss, Germany).

The number of Phospho-Histone-H3 positive cells was counted in the dermomyotomes and myotomes for both electroporated and uninjected contralateral (control) somites. Counts from pCAB-*Spry2* and pCAB-*Spry2*-Cterminus injected and uninjected somites were treated as paired readings (n=94). Statistical analysis was carried out using SPSS to calculate means and standard errors to confirm the significance of the observed differences (Wilcoxon test).

### RNA extraction and Northern blotting

RNA extraction followed by Northern blotting were carried out according to the methods described in [43]. Briefly, epithelial somites of embryos at HH10 (1.5 day) and HH27 (5 day) were dissected and washed in PBS and then transferred to lysis buffer containing guanidinum thiocyanate. Chloroform/isoamyl alcohol purification was carried out and RNA was precipitated in ethanol. For Northern blotting, 50 μg of total RNA was separated on 15% denaturating polyacrylamide gel electrophoresis (PAGE), stained with ethidium bromide/MOPS for 10 min and then visualized by UV trans-illuminator. RNA was transferred to five membranes (Hybond NX, Amersham Biosciences) then hybridized to ^32^P-labeled antisense probes (end-labelled with [γ-^32^P]ATP and T4 kinase) complementary to the mature mirRNA of gga-miR-21, gga-miR-23, gga-miR-27, gga-miR-122 and gga-miR-128 that cover the entire length of the miRNAs. Hybridization was carried out using EDC carbodiimide in 12.5 M 1-methylimidazole. Blots were pre-hybridised in UltraHyb Oligo (Ambion) and hybridised overnight at 37°C in a hybridisation oven. Membranes were washed twice for 30 min and then exposed at room temperature to Fuji Bass cassette 2040 (Fuji). Next, the membranes were stripped and hybridised with probe detecting the U6 small nuclear RNA as a control.

RNA extraction followed by Northern blotting were carried out according to the methods described in [43]. Briefly, epithelial somites of embryos at HH10 (1.5 day) and HH27 (5 day) were dissected and washed in PBS and then transferred to lysis buffer containing guanidinum thiocyanate. Chloroform/isoamyl alcohol purification was carried out and RNA was precipitated in ethanol. For Northern blotting, 50 μg of total RNA was separated on 15% denaturating polyacrylamide gel electrophoresis (PAGE), stained with ethidium bromide/MOPS for 10 min and then visualized by UV trans-illuminator. RNA was transferred to five membranes (Hybond NX, Amersham Biosciences) then hybridized to ^32^P-labeled antisense probes (end-labelled with [γ-^32^P]ATP and T4 kinase) complementary to the mature mirRNA of gga-miR-21, gga-miR-23, gga-miR-27, gga-miR-122 and gga-miR-128 that cover the entire length of the miRNAs. Hybridization was carried out using EDC carbodiimide in 12.5 M 1-methylimidazole. Blots were pre-hybridised in UltraHyb Oligo (Ambion) and hybridised overnight at 37°C in a hybridisation oven. Membranes were washed twice for 30 min and then exposed at room temperature to Fuji Bass cassette 2040 (Fuji). Next, the membranes were stripped and hybridised with probe detecting the U6 small nuclear RNA as a control.

## Abbreviations

AER: Apical Ectodermal Ridge; BMP: Bone Morphogenetic Protein; CEGMR: Center of Excellence in Genomic Medicine Research; CIPM: Center of Innovation in Personalized Medicine; DAPI: 4',6-DiAmidino-2-PhenylIndole; EMT: Epithelial to Mesenchymal Transition; ERK: Extracellular signal-Regulated Kinases; FGF: Fibroblast Growth Factors; GFP: Green Fusion Protein; HH: Hamburger-Hamilton; KACST: King Abdulaziz City for Science and Technology; MAPK: Mitogen-Activated Protein Kinases; Mgn: Myogenin; mir-RNA: microRNA; PSM: Pre-Segmented paraxial Mesoderm; RFP: Red Fusion Protein; RTK: Receptor Tyrosine Kinase; Shh: Sonic hedgehog; Spry2: Sprouty2; UTR: Un-Translated Region

## Authors’ contributions

A. M. and M. A. E. designed research; M. A. E. and K.G.-W. performed experiments; G. W. contributed new reagents/analytic tools; A. M., M. A. E., and K.G.-W. analysed data; A. M. and M. A. E. wrote the paper.

## Disclosures

The authors disclose no competing either financial or any other interests.

## Supplementary Material

Additional file 1 **Figure S1. *Spry2* expression at HH24 of chick embryo.** (A) Whole mount *in situ* hybridisation showing *Spry2* expression in somites, fore- and hindlimbs. (B) Transverse section at the level of the hindlimb bud showing *Spry2* strongly expressed in dorsal and ventral limb bud muscle masses. Magnifications: 22x in (A), 100x in (Ai). Click here for file

Additional file 2 **Figure S2. Expression of *Spry2* in somites and limb buds is closely associated with *Mgn*.** (A) Whole mount double *in situ* hybridisation of chick embryo at HH24 showing *Spry2* expression (in purple) combined with that of *Mgn* (in red) in somites and limb buds. (Ai) Transverse section at the level of forelimb bud showing *Spry2* expression overlapping with that of *Mgn* throughout the myotome and limb bud. (Aii) Transvers section through a myotome showing *Spry2* and *Mgn* overlapped expression. (Aiii) Frontal sections showing *Spry2/Mgn* expression in the myotomes. Magnifications: 18x in (A), 50x in (Ai), 200x in (Aii), 100x in (Aiii). Click here for file

## Declarations

Publication charges for this article were funded by the Centre of Excellence in Genomic Medicine Research (CEGMR), King Abdulaziz University, Jeddah, 21589, Kingdom of Saudi Arabia.

This article has been published as part of *BMC Medical Genomics* Volume 8 Supplement 1, 2015: Selected articles from the 2nd International Genomic Medical Conference (IGMC 2013): Medical Genomics. The full contents of the supplement are available online at http://www.biomedcentral.com/bmcmedgenomics/supplements/8/S1.
