# Supplementary figures and images for: Sprouty2 mediated tuning of signalling is essential for somite myogenesis
Source: BMC Med Genomics. 2015 Jan 15;8(Suppl 1):S8. doi: 10.1186/1755-8794-8-S1-S8 (PMC4315326; doi:10.1186/1755-8794-8-S1-S8)

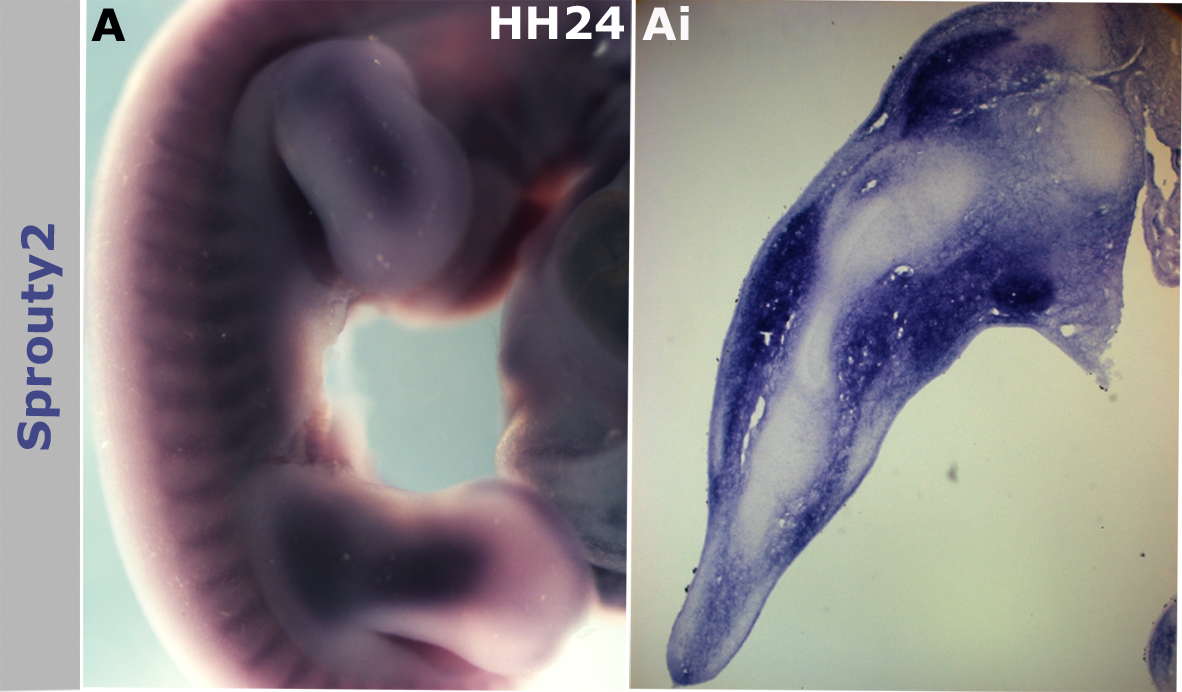

Supplement: Additional file 1 — Figure S1. Spry2 expression at HH24 of chick embryo. (A) Whole mount in situ hybridisation showing Spry2 expression in somites, fore- and hindlimbs. (B) Transverse section at the level of the hindlimb bud showing Spry2 strongly expressed in dorsal and ventral limb bud muscle masses. Magnifications: 22x in (A), 100x in (Ai). [file 1755-8794-8-S1-S8-S1.tif]

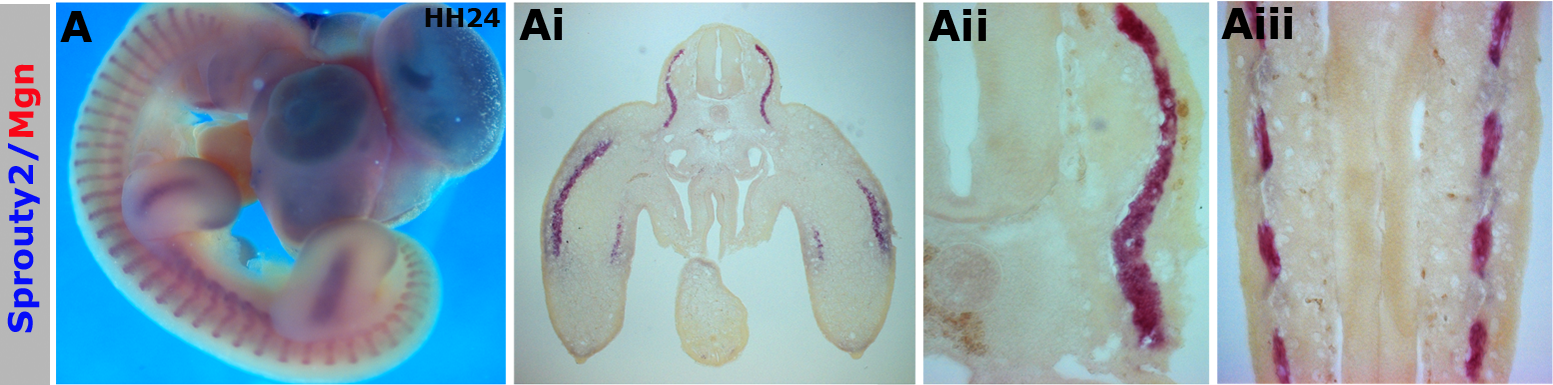

Supplement: Additional file 2 — Figure S2. Expression of Spry2 in somites and limb buds is closely associated with Mgn. (A) Whole mount double in situ hybridisation of chick embryo at HH24 showing Spry2 expression (in purple) combined with that of Mgn (in red) in somites and limb buds. (Ai) Transverse section at the level of forelimb bud showing Spry2 expression overlapping with that of Mgn throughout the myotome and limb bud. (Aii) Transvers section through a myotome showing Spry2 and Mgn overlapped expression. (Aiii) Frontal sections showing Spry2/Mgn expression in the myotomes. Magnifications: 18x in (A), 50x in (Ai), 200x in (Aii), 100x in (Aiii). [file 1755-8794-8-S1-S8-S2.tif]
